# Hospital readmissions following traumatic brain injury

**DOI:** 10.1186/cc12269

**Published:** 2013-03-19

**Authors:** A Boutin, K Francisque, L Moore, F Lauzier, X Neveu, A Turgeon

**Affiliations:** 1 Université Laval, Québec, Canada

## Introduction

Evaluating resource utilization is paramount in critically ill patients with traumatic brain injury (TBI), but little is known on readmissions after hospital discharge. We evaluated rates and determinants of unplanned readmission following TBI.

## Methods

We conducted a multicenter retrospective cohort study from April 1998 to March 2009. Data were obtained from a Canadian provincial trauma system, based on mandatory contribution from 59 trauma centres, and a hospital discharge database. Patients aged ≥16 years with TBI (ICD-9 or ICD-10 codes of 850-854 and S06, respectively) were included. Patients who died during the index hospitalization, who lived outside the province, who could not be linked with the hospital discharge database were excluded. We collected baseline and trauma characteristics, hospital admissions in the 12 months preceding index admission, and readmissions in the 12 following months. Primary outcome was unplanned readmission 30 days, 3 months and 6 months post discharge. We evaluated sociodemographic and clinical factors associated with readmissions using a logistic regression model.

## Results

Among 18,342 adult patients with TBI identified in the registry, 14,777 patients were included among which 2,363 had severe, 1,106 moderate and 11,308 mild traumatic brain injury. Most patients were young (mean age: 52 ± 23 years) and had no comorbidity (73.6%). Overall, 1,032 patients (7.0%) were readmitted within 30 days, 12.7% within 3 months and 17.6% within 6 months. At 30 days post discharge, 311 (30.1%) were readmitted for a complication. The median length of stay was 8 days (Q1 to Q3: 3 to 20). More than 10% of patients aged ≥75 years with ≥1 comorbidity or with ≥1 admission prior to index hospitalisation were readmitted. The severity of the TBI was not an independent predictor of readmission. Age, highest AIS, number of comorbidities, number of admissions prior to index hospitalization, level of index trauma center and discharge destination were associated with readmissions on multivariate analysis.

## Conclusion

Readmissions in the months following TBI are frequent, but were not found to be associated with the TBI severity. Further studies evaluating reasons for readmission are warranted in order to develop strategies to prevent such events.

